# Ti^3+^ Self-Doping of TiO_2_ Boosts Its Photocatalytic Performance: A Synergistic Mechanism

**DOI:** 10.3390/molecules29225385

**Published:** 2024-11-15

**Authors:** Mingqing Zhang, Manyu Liu, Keyi Han, Yingbin Liang, Xinyu Zhao, Lin Han, Jinnong Wang, Shifeng Wang, Yong Li

**Affiliations:** Key Laboratory of Plateau Oxygen and Living Environment of Tibet Autonomous Region, College of Science, Tibet University, Lhasa 850000, China; mingqingutibet@163.com (M.Z.); lmy15331930301@stu.utibet.edu.cn (M.L.); hankeyi@stu.utibet.edu.cn (K.H.); liangyingbin@stu.utibet.edu.cn (Y.L.); zhaoxinyutibet@163.com (X.Z.); hanlin@stu.utibet.edu.cn (L.H.); wangjinnong@stu.utibet.edu.cn (J.W.)

**Keywords:** photocatalyst, TiO_2_, Ti^3+^ self-doping, core shell, photodegradation

## Abstract

Pollution remains one of the most significant global challenges. Photocatalysis consists of a new organic pollutant removal technology, with TiO_2_ widely studied as a photocatalyst in the photocatalytic removal of water pollution. However, intrinsic TiO_2_ has the disadvantages of weak visible light absorption, low electron separation, and transmission efficiency, as well as few active sites. In this study, anatase-phase Ti^3+^ self-doped TiO_2_ (B-TiO_2_) with a core-shell structure was successfully prepared by forming an amorphous layer rich in oxygen vacancies (OVs) and Ti^3+^ defects on the TiO_2_ surface under a nitrogen atmosphere using NaBH_4_ as a chemical-reducing agent. The visible light absorption performance of the catalyst was notably improved when exposed to light irradiation. The bending of surface energy bands facilitated the separation of photogenerated electron-hole pairs, and the core-shell structure allowed the electron-hole pairs to be transported to the surface of the catalyst and participate in the reaction faster. We observed that 92.86% of Rhodamine B (RhB) was degraded in only 5 min, an increase of 2.73 times that of the degradation rate observed in commercial P25. With extraordinary stability, the photocatalytic efficiency of the catalyst remained at 96.2% after five degradation cycles.

## 1. Introduction

Pollution is one of the most severe challenges at present. Photocatalytic technology represents a novel approach to the removal of organic pollutants that requires only light to degrade organic pollution into water and carbon dioxide, with the advantages of mild reaction conditions, thorough purification, and universality, making it a truly environmentally friendly green technology [1,2,3,4,5,6,7,8]. The key to photocatalytic technology involves developing efficient photocatalysts. Titanium dioxide (TiO_2_) is regarded as a highly promising photocatalytic material within the realm of photocatalysts. This is primarily attributed to its wide availability, cost-effectiveness, exceptional chemical stability, non-toxic nature, and overall safety [9,10,11]. Common TiO_2_ crystal forms include anatase (3.2 eV), rutile (3.0 eV), and slate titanite (2.96 eV). Due to its wide bandgap, it is only sensitive to ultraviolet (UV) light, which makes up only 5 percent of the solar spectrum. Meanwhile, visible light, which accounts for the most significant proportion of solar energy, is not effectively utilized. The low efficiency of the electron-hole pair separation and transport, as well as a small number of reactive sites involved in the catalytic process, jointly limit the photocatalytic performance of TiO_2_ [12,13].

Haghighat et al. [14] explored the effects of different specific surface areas of TiO_2_ on photocatalytic performance. The researchers established a positive correlation between the specific surface area (BET) of the catalyst and the amount of active sites. This observation implies that there is a positive correlation between the increase of active sites and the BET, resulting in enhanced photocatalytic performance. Roberto et al. [15] successfully obtained Pt-doped TiO_2_ nanoporous catalysts using a simple two-step hydrothermal pathway, where the bandgap of Pt-TiO_2_ decreased to 2.15 eV, and there was a notable increase in the range of visible light absorption. However, the photocatalytic degradation of RhB for 180 min was only 63%. Chen et al. [16] first prepared black TiO_2_ through the high-pressure hydrogenation of TiO_2_ nanocrystals at 200 °C for 5 days, with a disordered layer on the surface resulting in a reduced band gap. Wang et al. [17] successfully synthesized Ti^3+^ self-doped TiO_2_ microspheres that exhibited exceptional stability. These microspheres possessed a bandgap of 2.89 eV, allowing for enhanced light absorption in the visible spectrum. This desirable property was achieved through the application of high-temperature hydrogen heat treatment. Ti^3+^ self-doping of TiO_2_ was also shown to introduce oxygen vacancies (OVs) and Ti^3+^ defects, affecting the energy band structure, shrinking the band gap, dramatically enhancing the light absorption performance, improving the photocatalytic performance of TiO_2_, enhancing photogenerated electron and hole separation efficiency, reducing compounding effects, and enhancing photocatalytic performance [17,18,19,20,21,22,23]. Thus, the modulation of TiO_2_ suffers from high preparation costs, high hazard factors, and low catalytic efficiency.

In this study, Ti^3+^ self-doped TiO_2_ (B-TiO_2_) with a core-shell structure was successfully prepared by using NaBH_4_ as a chemical reducing agent in a nitrogen environment, which is different from the conventional synthesis method of passing hydrogen at high temperature and high pressure with high safety. This self-doped core-shell structure, without introducing foreign elements, facilitates the separation and movement of electron(e^−^)-hole(h^+^) pairs generated by photolysis. Compared to commercial P25, B-TiO_2_ has a 1.5-fold increase in specific surface area and more active sites. In addition, the light absorption efficiency of B-TiO_2_ is greatly improved. Our designed and prepared B-TiO_2_ and its core-shell structure synergistically improved the catalytic performance of conventional TiO_2_ from various perspectives, such as light trapping, carrier separation, migration, etc. The degradation rate of RhB reached 92.86% after 5 min, which was 2.7 times higher than that of the catalytic performance of commercial P25. This study introduces a synergistic approach and provides a new technology for the synthesis of economical and efficient photocatalysts.

## 2. Results and Discussion

**Figure 1:**
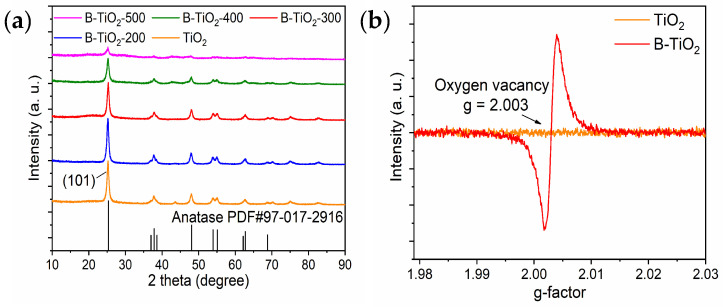
(**a**) XRD image and (**b**) electron paramagnetic resonance (EPR) of anatase TiO_2_ and B-TiO_2_.

Figure 1a presents the XRD spectra of TiO_2_ and B-TiO_2_, indicating that the precursor of B-TiO_2_ prepared by the hydrothermal reaction was anatase TiO_2_, and its corresponding standard PDF card number was PDF#97-017-2916. B-TiO_2_ was obtained by mixing TiO_2_ with the reducing agent in ball milling and then calcined in a nitrogen environment, and the results showed that there was no obvious shift in the position of the diffraction peaks as the calcination temperature increased, and the intensity of the diffraction peaks gradually decreased. Table 1 shows the comparison of the diffraction peak parameters of the samples before and after reduction, and the results show that the cellular parameters of the samples increased slightly after the reduction treatment, but they are still tetragonal crystal systems. The full width at half maxima of the diffraction peaks in the highest (101) plane becomes wider with the increase of calcination temperature. Combined with the current study, under high-temperature calcination in a nitrogen atmosphere, NaBH_4_, which exhibited strong reducing properties, could remove the O atoms in the surface layer of TiO_2_ to generate OVs and Ti^3+^ defects, forming an amorphous layer, and the amorphous layer of TiO_2_ and its encapsulated defect-free core ultimately formed a nuclear shell structure, and the size of the shell becomes thicker with increasing calcination temperature [24,25,26,27]. Since the shell structure itself does not produce XRD peaks, it will shield the X-ray absorption by the core of anatase TiO_2_, resulting in the phenomenon that the diffraction peaks of the prepared B-TiO_2_ are weakened with the increase of calcination temperature. As shown in Figure 1b, g = 2.003 of B-TiO_2_-300 was detected in the EPR experiments. By contrast, the existence of oxygen positions in anatase TiO_2_ was not detected, and this confirmed the presence of OVs in B-TiO_2_-300.

**Figure 2:**
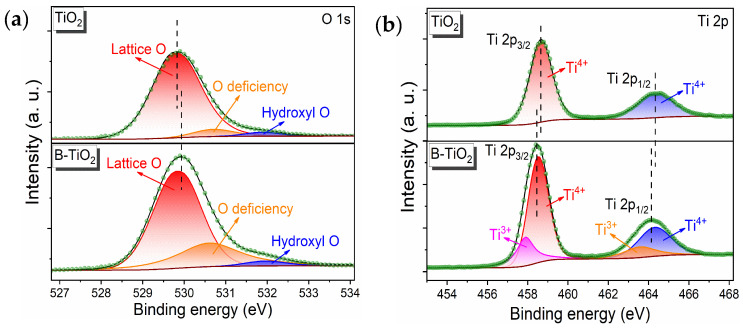
TiO_2_ and B-TiO_2_-300 (**a**), XPS O1s mapping (**b**), and XPS Ti2p mapping.

Figure 2 presents the XPS test results of B-TiO_2_-300 and TiO_2_, with Figure 2a showing the O1s fine spectra of elemental oxygen in B-TiO_2_-300 and TiO_2_. The peaks corresponding to Ti-O (lattice oxygen), oxygen defects (OVs introduced by calcination), and hydroxyl oxygen (water adsorbed on the surface) appeared in B-TiO_2_-300 at 529.8, 530.6, and 532.0 eV, respectively. The O1s nuanced spectrum of TiO_2_ also contained peaks corresponding to Ti-O, OVs, and hydroxyl oxygen. However, the peaks corresponding to its oxygen vacancies were significantly lower than those of B-TiO_2_-300, indicating that B-TiO_2_-300 obtained after high-temperature reduction with NaBH_4_ contained generated oxygen vacancies, which was in accordance with the EPR test outcomes. Figure 2b shows the Ti 2p fine spectra of the Ti elements in B-TiO_2_-300 and TiO_2_, indicating that B-TiO_2_-300 exhibited spikes representing Ti 2p_1/2_ and Ti 2p_3/2_ for Ti^4+^ at 464.1 eV, while the peaks referring to Ti^3+^ 2p_1/2_ and 2p_3/2_ appeared at 463.7 and 457.9 eV. The Ti 2p fine the spectrum of Ti elements in TiO_2_ only showed peaks that referred to Ti 2p_1/2_ and Ti 2p_3/2_ of Ti^4+^, demonstrating that B-TiO_2_-300 obtained by NaBH_4_ high-temperature reduction generated Ti^3+^, along with OVs generation. The photo-reactivity of the electronic structure was notably influenced by the presence of Ti^3+^ and OVs, resulting in an augmentation of the light absorption capacities. In addition, Ti^3+^ was in a defective state, which inhibited the recombining of photogenerated pairs of electron-holes and promoted the splitting of charges to improve conductivity [18,28].

**Figure 3:**
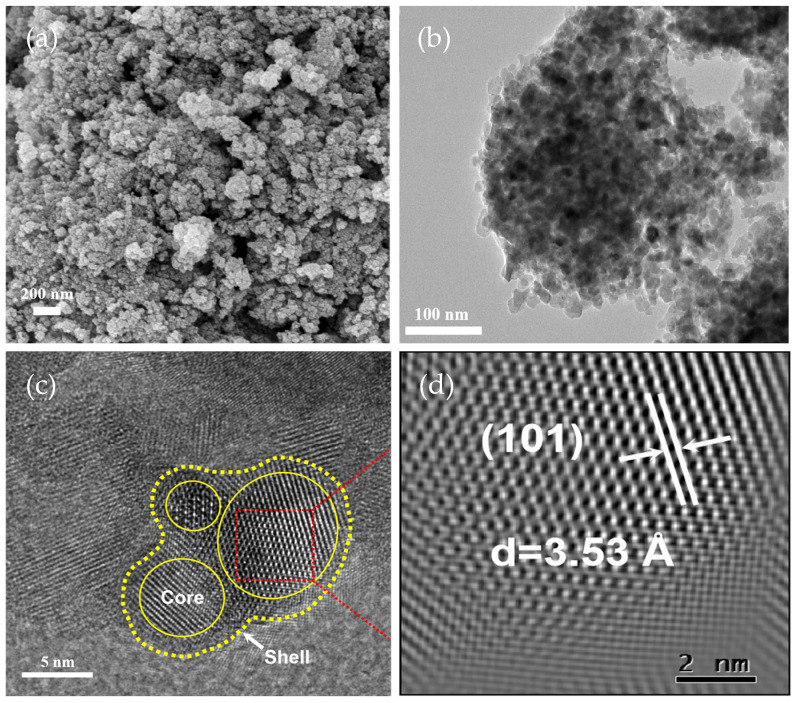
(**a**) SEM image, (**b**) TEM image, and (**c**,**d**) HRTEM of B-TiO_2_-300.

Figure 3a presents the B-TiO_2_-300 SEM image, indicating that the sample was composed of several tiny aggregated particles. TEM observations of the sample morphology also revealed that the sample’s particles varied in size and shape, as shown in Figure 3b. Ball milling was carried out during the preparation of this sample to uniformly mix NaBH_4_ and TiO_2_, and finer TiO_2_ particles were obtained, which contributed to the excellent catalytic efficacy of the B-TiO_2_-300 product. Figure 3c shows the HRTEM of B-TiO_2_-300, indicating that the internal lattice arrangement of the nucleus was ordered. Its lattice spacing value (3.53 Å) was also close to the corresponding value of the standard card of TiO_2_ (3.51 Å) (Figure 3d), and the outer crystal arrangement is disorderly due to the emergence of OVs and Ti^3+^ on the TiO_2_ exterior. Hence, the successful synthesis of B-TiO_2_, with an inner core and outer shell, was efficiently accomplished [29,30].

**Figure 4:**
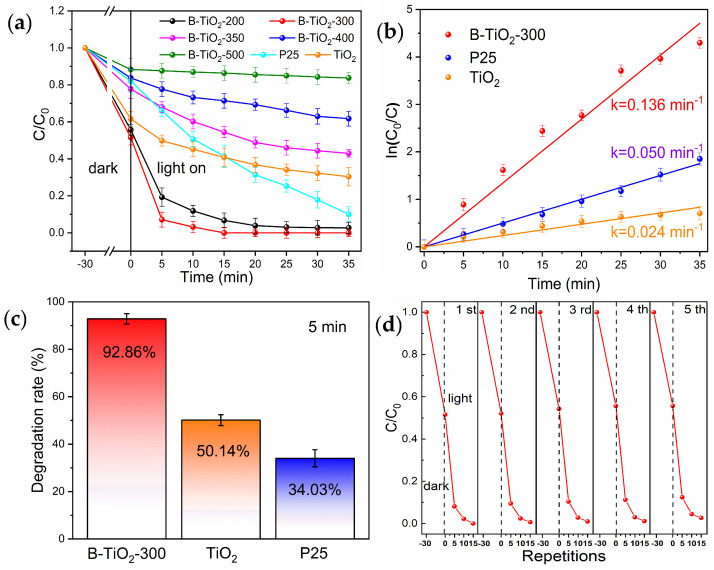
(**a**) Photocatalytic degradation, (**b**) first-order kinetics, (**c**) 5 min degradation rate, and (**d**) 15 min cyclic degradation of RhB by P25, TiO_2_, and B-TiO_2_-300.

Figure 4a displays the degradation curves of the experimental samples, revealing a trend in the photocatalytic breakdown efficiency of the samples that initially increased and subsequently decreased as the calcination temperature was raised. The performance of the photocatalytic breakdown of B-TiO_2_-300 was the best, which was significantly improved compared with the unreduced TiO_2_. After 5 min, the photocatalytic decomposition rate of RhB by B-TiO_2_-300 was 92.86% (Figure 4c), while the decomposition rates of unreduced TiO_2_ and commercial P25 were only 50.14% and 34.03%. The findings indicate that the photocatalytic degradation efficiency of B-TiO_2_-300 exhibited a significantly higher performance compared to the commercially accessible P25. The degradation rates of B-TiO_2_-300, P25, and TiO_2_ were compared, as shown in Figure 4b. According to the Langmuir-Hinshelwood model, the breakdown of RhB at low concentrations is seen as a first-order process. Hence, it is possible to simplify the degradation kinetics fitting calculations by employing a linear approach. Moreover, the reaction being examined follows the observed first-order reaction rate equation, Ln(C/C_0_) = −kt. During the initial phase of the reaction, k represented the apparent rate constant, and Ln(C/C_0_) was shown to be dependent on the duration of irradiation, denoted as t. The k-values of B-TiO_2_-300, P25, and TiO_2_ were 0.136, 0.050, and 0.024 min^−1^, respectively, which also showed that B-TiO_2_-300 had the strongest photocatalytic breakdown efficiency, which was substantially greater than untreated TiO_2_ and also significantly higher than commercial P25. The used B-TiO_2_-300 photocatalysts were recovered by centrifugal separation, and then repeated 15 min photocatalytic degradation tests were performed. The used B-TiO_2_-300 photocatalyst was recovered through centrifugal separation and then repeatedly tested for 15 min by photocatalytic degradation. It was observed that B-TiO_2_-300 had favorable stability, as evidenced by a photocatalytic degradation rate of 96.2% after undergoing five cycles. The exact experimental outcomes are depicted in Figure 4d.

**Figure 5:**
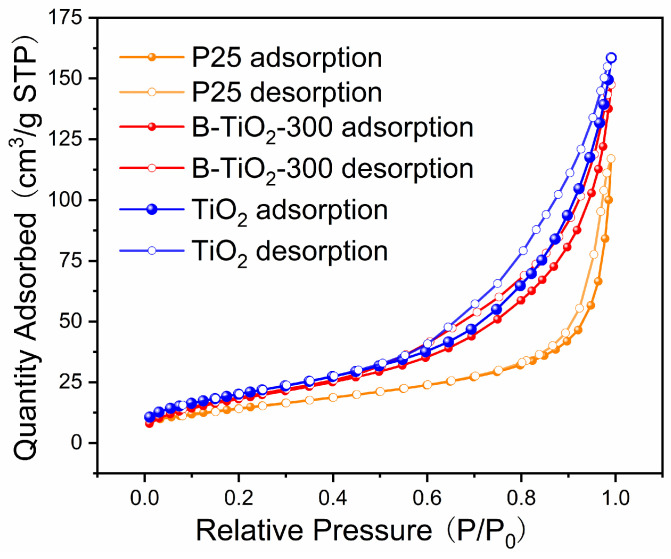
Nitrogen adsorption/desorption isotherm curves.

The type IV adsorption isotherms of B-TiO_2_-300 and P25 on nitrogen, which have H3-type hysteresis loops, are displayed in Figure 5. The BET values of the TiO_2_ and B-TiO_2_-300 samples were 75.0 and 69.9 m^2^/g, respectively, while the BET value of the commercial P25 sample was 46.7 m^2^/g. This indicated that B-TiO_2_-300 had a much larger area. Table 2 contrasts the specific surface extent of Ti^3+^ self-doped TiO_2_ in some recent research with that of B-TiO_2_-300. As the data demonstrates, B-TiO_2_-300 has a comparatively large specific surface area. The larger specific surface area of B-TiO_2_-300 can provide more active sites for the degradation reaction during the photocatalytic process, thus promoting photocatalytic performance.

**Figure 6:**
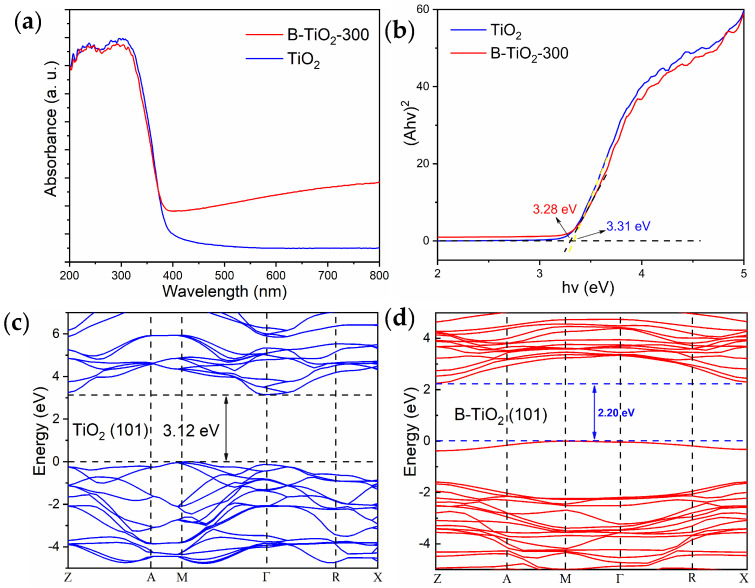
TiO_2_ and B-TiO_2_ (**a**) UV–visible diffuse reflectance (UDS), (**b**) TiO_2_ bandgap, (**c**) TiO_2_ DFT energy bands, and (**d**) B-TiO_2_ DFT energy bands.

As illustrated in Figure 6a, the UV-visible diffuse reflection line of TiO_2_ and B-TiO_2_-300 indicated that TiO_2_ exhibited minimal absorption within the visible light region. By contrast, the light utilization performance of B-TiO_2_-300 was significantly improved. The primary factor contributing to the improved light absorption properties of B-TiO_2_-300 is the existence of OVs and Ti^3+^ defects within the surface layer. The presence of these defects exerts a notable influence on the energy band structure, leading to the formation of defect energy levels, a decline in the valence band boundaries, and a narrowing of the band gap [27,39,40]. In contrast to TiO_2_, the light absorption edge of B-TiO_2_-300 did not exhibit a substantial enhancement, indicating that B-TiO_2_-300 was only partially reduced in the outer shell, and the inner core was still normal TiO_2_. Figure 6b illustrates the results of the experimental determination of the TiO_2_ and B-TiO_2_-300 band gap values, which were measured to be 3.31 eV and 3.28 eV, respectively. This value is acquired using the Kubelka-Munk theory. Additionally, Figure 6c displays the energy band diagram of TiO_2_, which is derived by DFT calculations. It is evident that TiO_2_ possesses a band gap of 3.12 eV, a value consistent with the experimental determination of TiO_2_ (Figure 6c). Figure 6d also shows the energy bands of B-TiO_2_-300 calculated using the same DFT method, which yielded a theoretical band gap value of 2.20 eV for ideal B-TiO_2_, and the band gap of B-TiO_2_-300 was significantly reduced compared with TiO_2_. The simulation results show that the defect layer of B-TiO_2_-300 has a lower band gap value, and the photogenerated electron-hole pairs are more easily separated. This accounted for B-TiO_2_-300′s enhanced visible-range photo-absorption performance, allowing the catalyst to produce more photogenerated electron-hole pairs under the same circumstances and participate in the photocatalytic process, thereby significantly enhancing photocatalytic performance.

**Figure 7:**
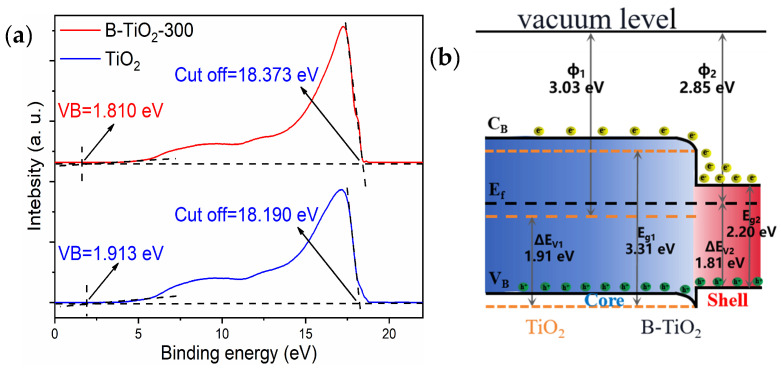
(**a**) Ultraviolet photoelectron spectra (UPS) of TiO_2_ and B-TiO_2_; (**b**) energy band diagrams.

Based on the results of UV photoelectron spectroscopy (UPS) tests, Figure 7 depicts the energy band alterations in TiO_2_ both before and after reduction. The valence band edge of B-TiO_2_-300 was 1.810 eV, whereas TiO_2_′s was 1.913 eV using UPS, as seen in Figure 7a. This difference in valence band edge between the two was the primary cause of B-TiO_2_-300′s bandgap reduction. The cut-off edge of the secondary electrons of B-TiO_2_-300 was 18.373 eV, while that of the TiO_2_ secondary electrons was 18.190 eV, and the ionization potentials IE required for the electron leaps of B-TiO_2_-300 and TiO_2_ were calculated as 2.85 and 3.03 eV, respectively, according to Equation (1). Figure 7b presents the energy band changes before and after TiO_2_ reduction combined with the above data and band gap values. The dashed line indicated the energy band diagram before the formation of homojunctions in TiO_2_, with the solid line indicating the equilibrium energy band diagram. The Fermi energy level position of B-TiO_2_ was higher, and the electrons flowed from B-TiO_2_ to TiO_2_ to form an accumulating layer for the electrons until the Fermi energy levels were equal. An electron depletion layer is located on the other side. They combine to produce a built-in electric field (qVD) that has the ability to accelerate the pair of e^−^ and h^+^ separation. After light irradiation, the lower the ionization potential, the easier the electrons could be excited and jump to produce pairs of e^−^ and h^+^. Furthermore, the effectiveness of the electron-hole pairs’ separation was enhanced under the action of the qVD. The greater the e^−^ and h^+^ concentration on the catalyst surface, the higher the photocatalytic degradation performance.

IE = hv − (E_0_ − E_f_) (1)

where the ionization potential, optical frequency, Planck’s constant, secondary electron cut-off edge, and valence band edge are represented, respectively, by the symbols IE, h, v, E_0_, and E_f_.

**Figure 8:**
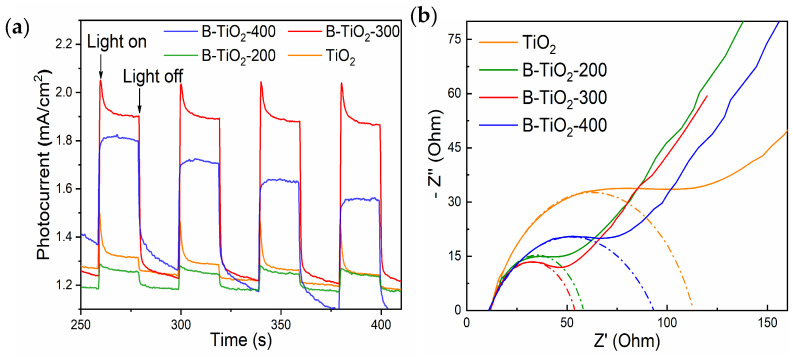
(**a**) Transient photocurrent response, and (**b**) electrochemical impedance Nyquist plots of TiO_2_ and B-TiO_2_.

Figure 8a shows the instantaneous photocurrent response curves of the experimental samples, and the higher photocurrent indicates the stronger separation of photogenerated electron-hole pairs. From the figure, it can be seen that the photocurrent of B-TiO_2_-300 is the highest and much larger than that of TiO_2_, which indicates that the photogenerated electron-hole in B-TiO_2_-300 is easy to separate, which is also an important reason for the enhancement of photocatalytic degradation performance of B-TiO_2_-300. Figure 8b shows the electrochemical impedance Nyquist plots of the experimental samples. The migration performance of photogenic electrons and holes is reflected by the curvature radius of the curved arc. A lower curve radius indicates greater migration performance, whereas a larger radius indicates poorer migratory performance. Hence, it can be observed that the migration performance of photogenerated e^−^ and h^+^ was most optimal in the B-TiO_2_-300 material. Enhancing the migration efficiency of photogenerated e^−^ and h^+^ can effectively mitigate the recombination of these charge carriers, hence facilitating the enhancement of photocatalytic performance. On the other hand, photogenerated electrons and holes can migrate faster to the catalyst surface to participate in the reaction, which simultaneously improves the overall photocatalytic performance. Based on recent research findings [41,42,43,44], the enhanced abilities of electron and hole migration and segregation in B-TiO_2_-300 can be primarily attributed to the development of an amorphous layer containing an abundance of OVs and Ti^3+^ defects within the topmost layer of TiO_2_. This amorphous layer is formed by the introduction of NaBH_4_, which results in a homogeneous core-shell structure with the TiO_2_ core.

**Figure 9:**
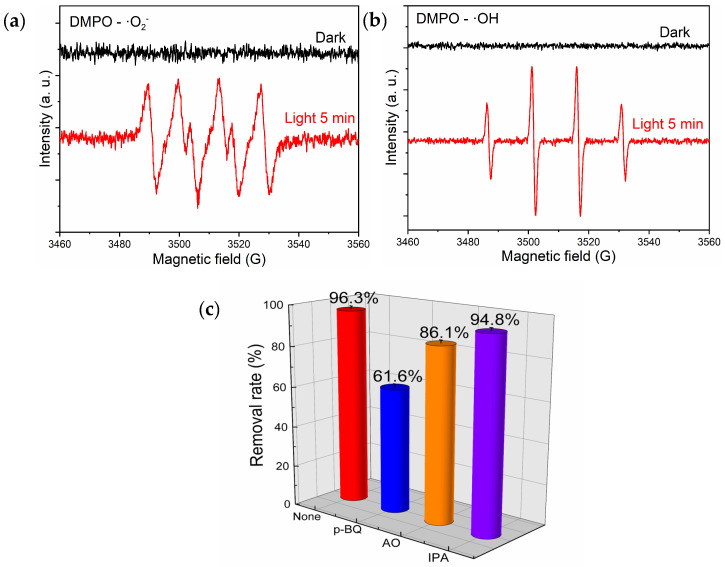
EPR spectra of the DMPO trapping radicals on B-TiO_2_-300: (**a**) DMPO-·O_2_^−^, (**b**) DMPO-·OH, and (**c**) degradation of RhB by B-TiO_2_-300 and the addition of different scavengers: p-benzoquinone (p-BQ), ammonium oxalate (AO), and isopropyl alcohol (IPA), after visible light irradiation (λ > 300 nm, 30 s).

The experiment involved the utilization of DMPO as a radical scavenger to conduct electron paramagnetic resonance (ESR) analysis on B-TiO_2_-300. The purpose was to investigate the generation of ·O_2_^−^ and ·OH radicals in the presence of non-light and light illumination conditions. The obtained results are presented in Figure 9a,b. The figure indicates that no ESR signal was detected in the absence of light and that four distinct peaks with an intensity ratio of 1:1:1:1 were seen in the a-figure following exposure to visible light. These peaks were brought on by DMPO’s capture of ·O_2_^−^. In the meantime, the b-figure displays four distinct peaks with an intensity ratio of 1:2:2:1, caused by the capture of ·OH by DMPO, suggesting that under light irradiation, the B-TiO_2_-300 surface produced ·O_2_^−^ and ·OH radicals. Further capture experiments were conducted in order to examine the active species that were crucial to the photocatalytic degradation process and to learn more about the photocatalytic degradation mechanism of RhB by B-TiO_2_-300. P-benzoquinone (p-BQ), ammonium oxalate (AO), and isopropanol (IPA) were selected as scavengers of superoxide radicals (·O_2_^−^), hydroxyl radicals (·OH), and holes (h^+^), respectively, and the study illustrated the inhibitory effects of several radical scavengers on the degradation of RhB by B-TiO_2_-300, as depicted in Figure 9c [45]. Including the p-BQ scavenger greatly lowered the breakdown rate of RhB, adding the AO scavenger slightly reduced the degradation rate of RhB, and the extra of the IPA scavenger barely impacted the breakdown rate of RhB. The findings of this study suggest that the presence of ·O_2_^−^ had a notable impact on the degradation of RhB, whereas the involvement of ·OH in the degradation process was relatively low. Additionally, it was observed that h^+^ had minimal influence on the degradation of RhB.

## 3. Materials and Experiment

### 3.1. Materials

Shanghai Aladdin Biochemical Technology Co., Ltd. provided the sodium hydroxide (NaOH), ethylene glycol ((CH_2_OH)_2_), and tetrabutyl titanate (C_16_H_36_O_4_Ti). (Shanghai, China), Tianjin New Technology Industrial Park Chemo Chemical Reagent Co., Ltd. (Tianjin, China) and Xilong Scientific Co., Ltd. (Chengdu, Sichuan, China), respectively. NaBH_4_ had been purchased from FUCHEN Co., Ltd. (Tianjin, China). Each of the previously stated reagents was used without any prior pre-treatment and had an analytical purity. The water employed in the experimental procedure was prepared in-house and was deionized, originating from the laboratory.

### 3.2. Synthesis of TiO_2_

Tetrabutyl titanate was added to 1 M sodium hydroxide liquid and agitated at ambient temperature for 10 min. Tetrabutyl titanate was sonicated for 5 min to homogeneously disseminate in the solution. Following that, the solution underwent the addition of ethylene glycol and was subjected to stirring for a duration of 10 min. The gathered white liquid subsequently went to a 150 mL reaction kettle, which was positioned in a heated oven and held at 150 °C for 12 h. The precipitate was next immersed in 0.1 mol/L hydrochloric acid for 6 h and crashed by filtration with ultrapure water a few times until the pH value reached neutral. The resulting precipitate was subjected to a drying process at a temperature of 60 °C for 10 h. Subsequently, it underwent a calcination process at a temperature of 400 °C for a duration of 4 h, resulting in the production of TiO_2_ with a significantly elevated specific surface area.

### 3.3. Synthesis of B-TiO_2_

To uniformly mix TiO_2_ with NaBH_4_, 200 mg of TiO_2_ and 18.94 mg of NaBH_4_ were placed into a ball mill (Li-Chen, LC-PTG-6) for 5 min. The obtained powder was then transferred into a square furnace, and the container was put in a tube heater (Kocrystal GSL-1700X-II, Hefei Kejing Material Technology Co., Ltd, Hefei, China) and calcined at 300 °C for 3 h under a nitrogen-filled environment. After that, the tube furnace was subjected to heating at a rate of 10 °C per minute. The resulting powdered product underwent several washes using ethanol and deionized water, followed by drying at a temperature of 60 °C for a duration of 10 h. The final product was denoted B-TiO_2_-300, with the number 300 indicating the calcination temperature of the tube furnace, and samples B-TiO_2_-200, B-TiO_2_-350, B-TiO_2_-400, and B-TiO_2_-500 were prepared by changing only the calcination temperature in the same manner.

### 3.4. Analytical Test Method

The X-ray diffraction (XRD) technique was utilized to ascertain the crystalline composition of the specimens. A Bruker D8 ADVANCE X-ray diffractometer, equipped with a copper (Cu) target, was employed for this purpose. A transmission electron microscope (TEM, Thermo Fisher, model FEI Talos F200X) and the field-emission scanning electron magnifying glass (SEM, Zeiss, model Gemini 300, Oberkochen, Germany) were utilized to observe the microscopic structure of the samples. The UV-Vis absorption spectra were acquired with a UH4150 UV-Vis spectrophotometer produced by Hitachi (Tokyo, Japan). The wavelength range for the measurements was adjusted to 200–800 nm. A McASAP 2460 instrument measured the total area of the surface and pore diameter (BET), and the materials were heated at 200 °C for 6 h, with nitrogen gas used for adsorption and degassing. The samples’ elemental composition and valence states were examined using an Escalab 250XI X-ray photoelectron spectrometer (XPS) manufactured by Thermo Fisher, Waltham, MA, in the United States. EPR (Bruker, Karlsruhe, Germany: A300-10/12) was performed using DMPO and TEMP as the free radicals and oxygen vacancy trapping agents, respectively. An electrochemical workstation (CHI700E series, universal dual constant potential meter) was used to measure the transient photocurrent response and impedance, and a UV–visible spectrophotometer (UV-DRS, L5S) was used to study the optical absorption spectra.

### 3.5. Photocatalytic Performance Test

The produced photocatalysts were assessed for their ability to degrade organic pollutants using Rhodamine B (RhB) as a simulated substance. A 20 mg/L RhB fluid consisting of 100 mL was poured into a 400 mL beaker, and 20 mg of the test sample was placed in it and sonicated for 30 min at ambient temperature. The entire sonication procedure was carried out in a dark room. Then, the beaker was set under the illumination of a sunlight simulator (Solar-500Q-Xenon lamp, 200–1200 nm, Newbie, Beijing, China), and the brightness of the light source was adjusted so that the light intensity at the liquid surface of the beaker was 600 W/m^2^. The photocatalytic degradation experiment was conducted for 60 min, with 4 mL of the reaction solution obtained from the centrifuge tube every 5 min. The supernatant was taken after centrifugation, and a UV-visible spectrum analyzer was used to determine the absorbance of the residue, which was recorded as An, while the 20 mg/L RhB solution was recorded as A_0_. The recorded value for the absorbance of the supernatant following the process of centrifugation was denoted as A_1_, with the light source’s wavelength set at 554 nm. The RhB concentration in the supernatant was recorded as C_n_, C_0_ indicated the amount of 20 mg/L RhB solution, and C_1_ was the concentration of centrifugal supernatant after ultrasonication. The relationship between absorbance and concentration was calculated according to:

C_n_/C_0_ = A_n_/A_0_

### 3.6. Theoretical Calculations

The researchers employed the Vienna Ab-initio simulation package (VASP) code to perform Density Functional Theory (DFT) computations [45,46,47,48]. The total energy estimates utilized a plane wave cut-off energy of 400 eV. In this study, the representation of ion-electron interactions utilized ultrasoft pseudopotentials, which were incorporated into the projector-augmented wave (PAW) approach [49]. The exchange-correlation function utilized in this study was the Perdew-Burke-Ernzerhof (PBE) functional, which falls under the category of generalized gradient approximation (GGA) [49,50]. The process of integrating over the Brillouin zone was approximated by summing a carefully selected set of k-points. This summation was performed using the Monkhorst-Pack method, with a grid size of 2 × 2 × 1 [51]. The geometries were iteratively adjusted until the energy reached a convergence threshold of 1 × 10^−5^ eV per atom, and the forces reached a magnitude of 0.02 eV per Ångstrom. A p(1 × 3) supercell was used to model the (101) surface. To mitigate the interactions between the slabs, a vacuum area of 15 Å was established along the z-axis to create separation between them.

## 4. Conclusions

In summary, we obtained self-doped TiO_2_ with a high specific surface area by calcining TiO_2_ mixed with the NaBH_4_ reductant under a nitrogen atmosphere, which demonstrated outstanding safety compared with traditional H_2_ and the metal reduction method. An amorphous layer rich in oxygen vacancies (OVs) and Ti^3+^ defects formed on the TiO_2_ surface formed a core-shell structure with anatase titanium dioxide nuclei, and a homogeneous junction between the nuclei and shells lowered the bandwidth of B-TiO_2_-300 and introduced impurity energy levels into the band gap. This greatly improves the absorption efficiency of B-TiO_2_-300 for visible light and also enhances the separation and mobility properties of photogenerated electron-hole pairs in B-TiO_2_-300. These changes significantly improve the photocatalytic degradation performance of B-TiO_2_-300. Notably, the performance of B-TiO_2_-300 surpassed that of the commercially available P25, with B-TiO_2_-300 degrading 92.86% of RhB within 5 min, compared to a degradation rate of only 34.03% for commercial P25.

## Figures and Tables

**Table 1:** Cell parameters of the samples and full width at half maxima of the diffraction peak (101) plane.

Samples	a = b	c	α = β = γ	FWHM (2θ)
TiO_2_	3.778	9.495	90°	0.620
B-TiO_2_-200	3.782	9.498	90°	0.628
B-TiO_2_-300	3.783	9.499	90°	0.636
B-TiO_2_-400	3.781	9.503	90°	0.697
B-TiO_2_-500	3.783	9.504	90°	0.711

**Table 2:** Comparison of specific surface area.

	Specimens	BET (m^2^/g)	Photocatalytic Degradation	Efficiency	Literatures
1	B-TiO_2_-300	69.9	RhB (20 mg/L)	20 mg catalyst, 20 min, 98.6%	this text
2	P25	46.7	RhB (20 mg/L)	20 mg catalyst, 20 min, 68.6%	this text
3	The reduced TiO_2_ nanoparticles	35.3	RhB (20 mg/L)	100 mg catalyst, 300 min, Approximately 80%	[31]
4	Ti^3+^-doped TiO_2_	49.4	MB (10 mg/L)	30 mg catalyst, 20 min, 97.2%	[21]
5	Black TiO_2_	42.4	MB (10 mg/L)	400 mg catalyst, 20 min, 82.2%	[23]
6	Ti^3+^ self-doped TiO_2_	37.7	RhB (10 mg/L)	50 mg catalyst, 30 min RhB catalyst 100%	[32]
7	Ti^3+^ self-doped TiO_2_	54.4	RhB (10 mg/L)	70 mg catalyst, Approximately 99%	[33]
8	CTP/TiO_2_	49.4	RhB (10 mg/L)	100 mg catalyst, 6 h 87.7%	[34]
9	TNS/WS_2_-0.10	76.1	RhB (20 mg/L)	20 mg catalyst, 90 min 100%	[35]
10	TiO_2_/g-C_3_N_4_/RGO-2	74.05	RhB (10 mg/L)	50 mg catalyst, 60 min 99.1%	[36]
11	Chl-Au_25_/P25 (uc)	-	RhB (30 mg/L)	30 mg catalyst, 50 min 100%	[37]
12	Ti/TiO_2_(1/10)	-	RhB(50 mg/L)	50 mg catalyst, 180 min 100%	[38]

## Data Availability

Data are contained within the article.
